# Supplementary material for: Optically-biased Rydberg microwave receiver enabled by hybrid nonlinear interferometry
Source: Nat Commun. 2025 Oct 16;16:8975. doi: 10.1038/s41467-025-63951-9 (PMC12531335; doi:10.1038/s41467-025-63951-9)
Supplement: Supplementary file 1 — Supplementary Information [file 41467_2025_63951_MOESM1_ESM.pdf]

Supplementary information for: "Optically-biased Rydberg microwave receiver enabled by hybrid nonlinear interferometry"

S.1 SUPPLEMENTARY DISCUSSION ON THE CHOICE OF FREQUENCIES FOR PROCESSING

The choice of beating frequencies for processing is dependent more on the noise characteristics of an implementation, rather than the properties of atoms, as typically the receiving bandwidth can be tuned around the Rydberg transition, taking advantage of the detunings of the various optical fields that take part in the process.

In our case, at the signal photodiode below 500 kHz, we observe a vast increase in the noise that we attribute to electronic (flicker) noise and power fluctuations of the probe field, due to laser frequency fluctuation transduced to probe beam power fluctuation via narrow EIT features. Tailoring detection at higher frequencies enables getting rid of that noise in the detection band. We set the central frequency to be detected at $\Delta = 1.8$ MHz, as shown in Sup. Fig. 1.

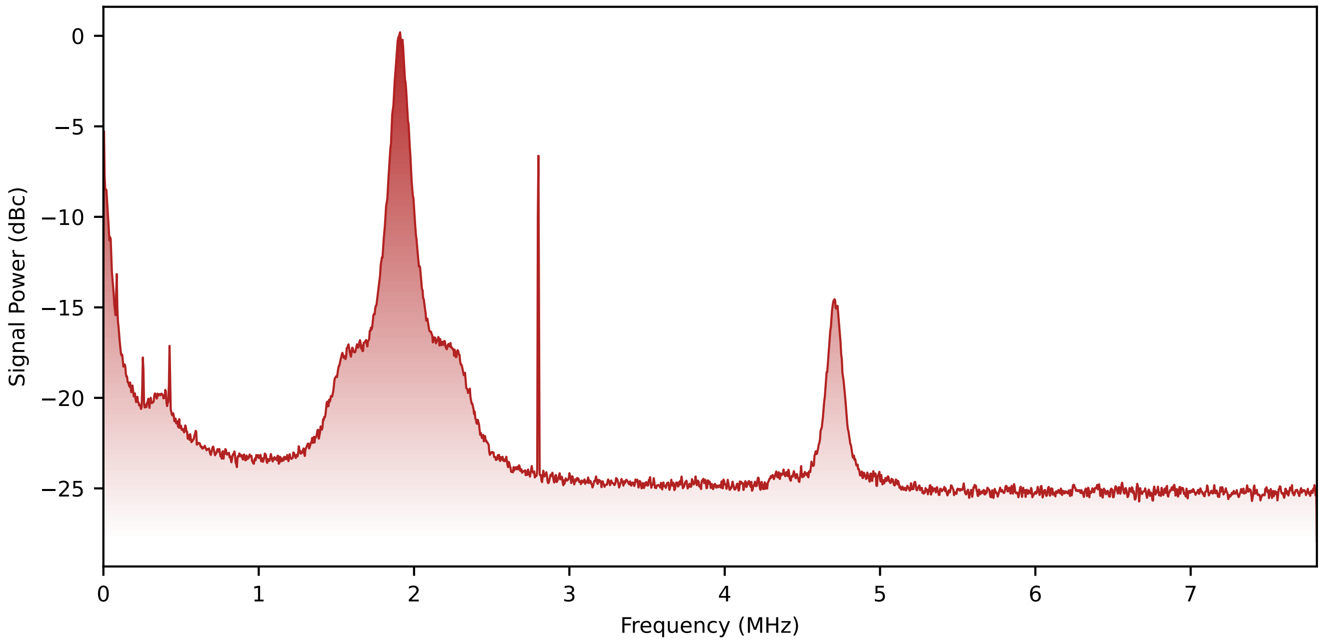

Supplementary Figure 1. The frequency readout from the signal photodiode. The signal is registered at $\Delta = 1.8$ MHz. Below 500 kHz, the noise from probe field power fluctuation is dominant, while the rest of the spectrum is shot noise limited. The peak at 4.6 MHz is a leak from the reference setup, present only due to electrical connections between sources (yet, this is why it is important to set $\Delta \neq \Delta'$). The narrow peak at 2.8 MHz is a superhet-type detection occurring due to the two broad signals beating with each other. In the processing, an IQ mixer is set at the central $\Delta = 1.8$ MHz frequency with 1 MHz bandwidth (and strong low-pass filtering), so that the other signals do not enter the detection. The spectra are estimated from experimental data using Welch's method.

Apart from the low-frequency noise, other sources of noise can be frequency-filtered by choosing the right frequencies for demodulation at IQ mixers. In our implementation, we observed a leak from the reference frequency source, present at $\Delta' = 4.6$ MHz (see Sup. Fig. 2 for the signal registered at the reference photodiode). This interference is the result in the specific electrical connections in our system, designed so that we could easily switch between optical-bias and superhet detection in the same setup, so this is not an inherent flaw of the optical-bias method. Additionally, this leads to another component of beating between both registered signals at 2.8 MHz. Nevertheless, both of these interferences can be filtered out via proper choice of central frequencies and bandwidths for demodulation.

As mentioned, the detection bandwidth can be shifted with tuning of the optical fields taking part in the optical-bias process. In our case, we found experimentally that the most crucial tool for this shift is the Δ_{5D} detuning. We found out that for low Δ frequencies the optimal working point is when $\Delta_{5D} = -\Delta$, and thus set the $\Delta_{5D} = -1.8$ MHz. This is however, not true for larger detunings, as demonstrated below in the consideration of bandwidth (Supplementary Section S.4).

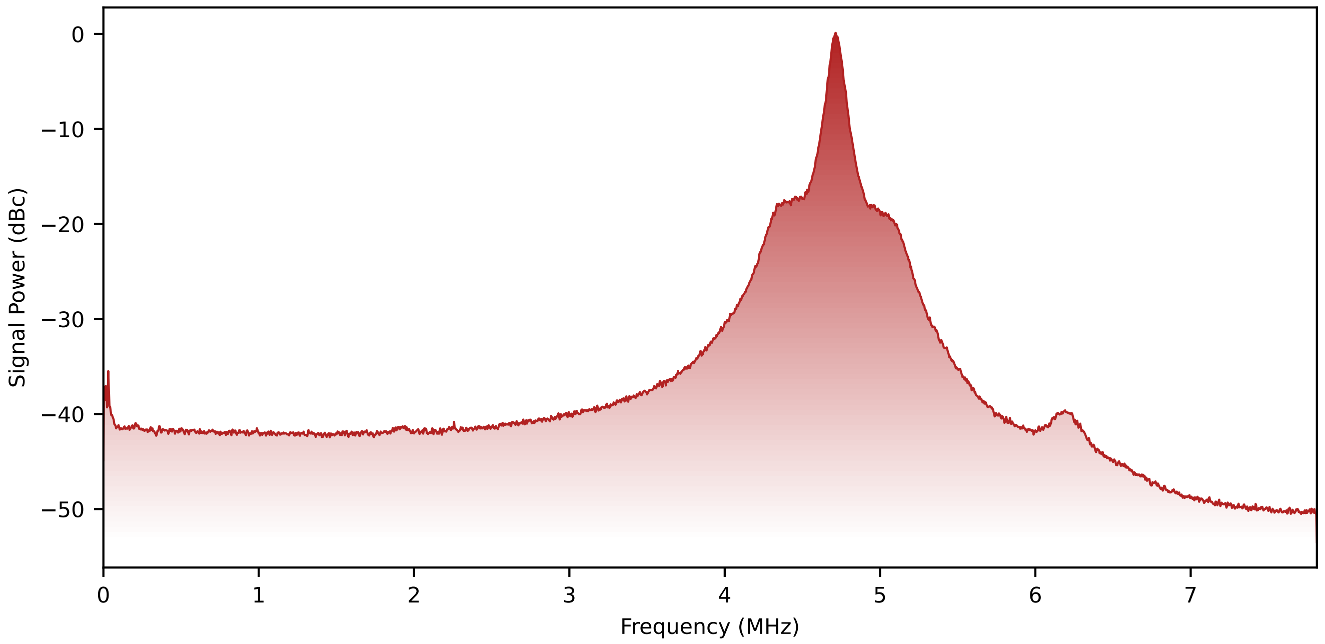

Supplementary Figure 2. The frequency readout from the reference photodiode. The reference signal is registered at $\Delta' = 4.6$ MHz. At the very low frequencies, there is some DC offset and noise from amplitude fluctuation. The frequencies > 6 MHz are low-pass filtered to cut off higher-order modulation artifacts. One such artifact can be seen at 6.2 MHz due to the aliasing effect. The spectra are estimated from experimental data using Welch's method.

S.2 SUPPLEMENTARY DISCUSSION ON THE SHOT NOISE LEVEL

In the typical cases of both superhet detection and optical-bias detection, the factor limiting the readout of the measured RF field is the shot noise of the probe optical field (provided that the photodiode detector is sufficiently low-noise). While in the case of superhet, this limitation has been studied in literature [1], we present the measurements reinforcing this claim in the case of the optical-bias method.

In the optical detection noise, the total noise may be composed of a part linear in the optical power – the shot noise, a constant part – typically electronic noise, and a quadratic part – present due to technical noise in the optical signal itself. In our work, we avoid both electronic noise and technical noise by shifting the detection frequency to the RF range.

The results are presented in Sup. Fig. 3. Concerning the working point, in which all the measurements in the main manuscript were taken, we increase and decrease the power of the probe field with the MW field switched off. Noise levels of the readout (after the processing via IQ mixers) were measured, and we observe that these levels follow the linear relation, from which we obtain a fit with directional coefficient $a = 0.84$ and residual coefficient $b = 0.17$. The latter of the coefficients can be interpreted as non-shot noise (primarily electronic noise), constituting 17% of the total noise registered. Consequently, the shot noise of the probe optical field at the readout constitutes 83% of the total noise, and thus the measurement can be considered shot noise limited. Most importantly, we observe that the dependence is primarily linear, supporting the claim that shot noise is the main component in this parameter range.

S.3 SUPPLEMENTARY DISCUSSION ON THE LONG-TERM STABILITY

The universal measure of long-term stability of an oscillator is the Allan deviation. In the main text, we present the power spectrum of the compensated signal z_C with a 100 ms time window, showing it is essentially Fourier limited. To study the long term stability, we collected the z_C signal for $\tau_{\max} = 100$ s, estimated its phase as $\arg(z_C)$ and computed the overlapping Allan deviation $\sigma(\tau)$ using the *AllanTools* (version 2024.06) Python software package [2].

We plot the Allan deviation in Sup. Fig. 4. The plot clearly shows that the signal phase fluctuations are dominated by the white phase noise (i.e. shot noise in our case) up to 100 ms averaging. The stability is the best at $\tau = 1.4$ s and amounts to 2.1 Hz. Longer averaging increases the deviation due to frequency random walk, which is most likely due

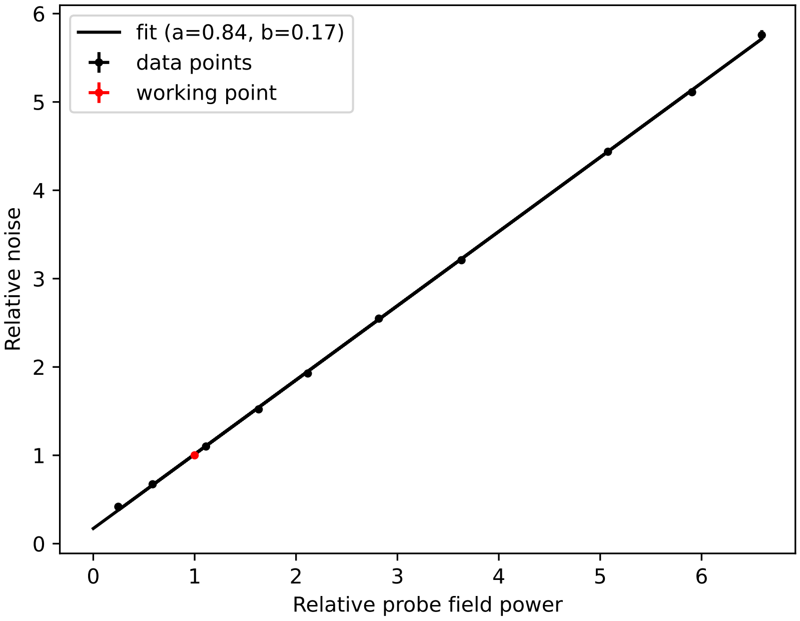

Supplementary Figure 3. The relation between the noise at the readout and the power of the probe field used for the detection. Both units are presented relative to the working point, which was chosen experimentally to provide the best signal-to-noise ratio. The linear fit reveals directional coefficient $a = 0.84$ and residual coefficient $b = 0.17$, the latter of which is interpreted as non-shot added (electronic) noise. The uncertainties shown are standard error over $n = 8$ repetitions for each data point.

to the relative stability of FPGA and MW PLL clocks. The intermediate regime is dominated by other kinds of noise, likely resulting from the interferometric stability of the setup, i.e. acoustic noise. Different contributions are plotted as results from the fit of a general power-law combination to the experimental data, $\sigma_\tau(\tau) = A/\tau + B/\sqrt{\tau} + C + D\sqrt{\tau}$.

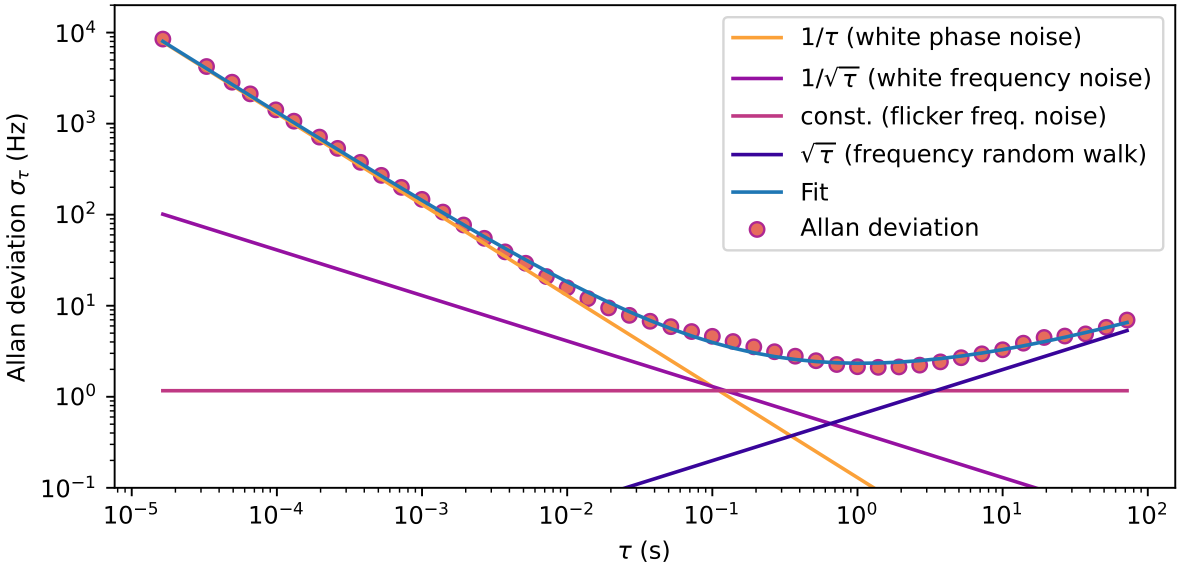

Supplementary Figure 4. Allan deviation $\sigma(\tau)$ for the compensated all-optical superhet signal z_C (driven by a monochromatic signal $\mathcal{E}$) as a function of averaging time τ. The experimental dependence is fitted with a sum of power-law dependences, with each component also drawn individually.

S.4 SUPPLEMENTARY DISCUSSION ON THE BANDWIDTH

The choice of bandwidths and central frequencies for demodulation was dictated by the demonstration of the best performance in terms of sensitivity. However, in specific applications, a wider bandwidth may be considered at a trade-off of some of the sensitivity.

To demonstrate that and analyse the limitations of bandwidth due to inherent atomic properties, we perform an additional measurement. We measure the resonant MW frequency at $\omega_0 = 13916.1$ MHz (it should be noted that this measurement was done in a different vapour cell, 25 mm optical length, and rubidium-87, which may have amounted to a small shift of the central frequency compared to the main text), set the reference frequency far to avoid interference in the bandwidth (we chose 13939.1 MHz) and perform point by point measurement, while changing the detuning Δ of the signal field.

The results are presented in Sup. Fig. 5. To obtain these results of a shifted bandwidth, we applied $\Delta_{\mathrm{5D}} = -8$ MHz. An asymmetrical band shape can be observed with 5.8 MHz FWHM bandwidth and centre at $\Delta = 9.5$ MHz. The asymmetry may be attributed to the bright resonances taking part in the process, similarly to the MW-to-optical conversion [3], while the centre shift larger than 8 MHz indicates that the $\Delta_{\mathrm{5D}} = -\Delta$ relation, observed for smaller detunings, is no longer strictly followed.

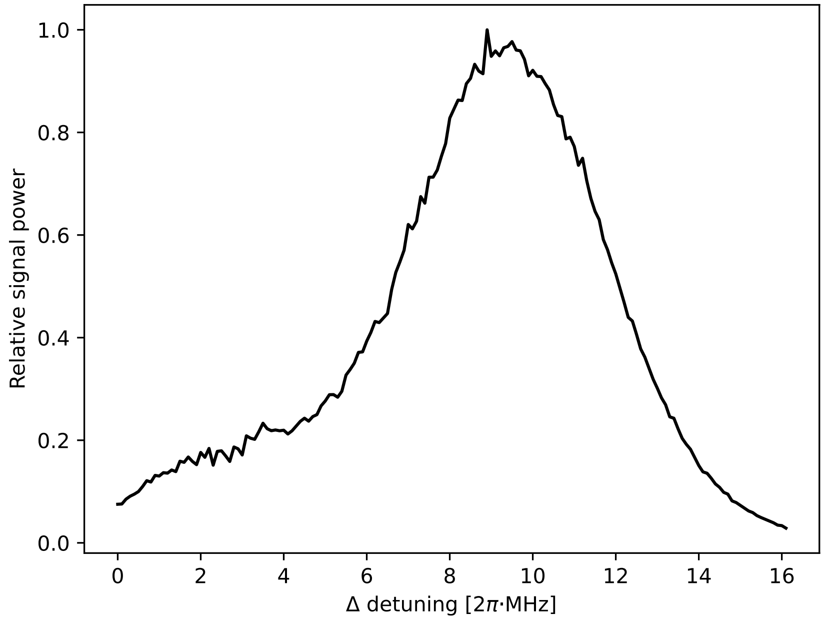

Supplementary Figure 5. The registered band of optical-bias detection. The $\Delta_{\mathrm{5D}} = -8$ MHz was set to shift the band from low frequencies. We estimated the FWHM bandwidth to be 5.8 MHz, while the centre of the band is shifted to $\Delta = 9.5$ MHz.

S.5 SUPPLEMENTARY DISCUSSION ON THE RECEIVER'S RECEPTION PATTERN

The transduction of the MW signal to the optical domain, in general, depends on the angles of the optical and MW fields involved in the process. To account for this, we must consider the spatial dependence of the signal imprinted onto the probing optical field, particularly its spatial phase dependence. The optical signal accumulates along the interaction region (where all beams intersect) and, due to spatial averaging – or more precisely, phase matching – this spatial variation affects the transduction efficiency. In the typical case of MWs much longer than the beam widths $\lambda_{MW} \gg w_0$, the phase varies only along the probe beam. It becomes important when the spatial period of the phase $1/\delta k_z$ (where δk_z is the phase mismatch along the optical propagation axis) is comparable to the length of the interaction region L. This leads to a reception (antenna-like) pattern of the receiver. This applies to both the superheterodyne and optical-bias methods.

In the superheterodyne method, since the phases of the probe and coupling beams cancel out, the spatial phase is determined only by the difference in wavevectors of the signal and LO MW fields $\delta k_z = k_z^{\mathcal{E}} - k_z^{LO}$, which is zero in the typical case of copropagating fields.

In the optical-bias method, the spatial phase includes contributions from the biasing optical fields and the signal MW field. For large optical beams, i.e. when the Rayleigh range z_R is larger than the length L, the mismatch can be approximated as $\delta k_z = k_z^{C1} + k_z^{\mathcal{E}} - k_z^{C2} - k_z^{D}$. For small beam widths ($z_R \sim L$), the situation becomes more complex, as the phase mismatch also depends on the transverse coordinates.

To calculate the reception pattern, we can evaluate the phase-matching integral multiplied by the factor arising from projecting the signal MW field onto the polarisation set by the polarisations of the optical fields. In the most common cases of collinear and circularly polarised beams, we can leverage the axial symmetry and parametrise the pattern by a single angle θ:

$$\eta(\theta) \propto \cos\left(\frac{\theta}{2}\right)^2 \int_0^L \mathrm{d}z \int_0^{2\pi} \mathrm{d}\varphi \int_0^\infty \rho \mathrm{d}\rho \Theta_\theta(\rho, \varphi, z), \tag{1}$$

where Θ_θ is the product of complex spatial distributions of all fields and conjugate fields taking part in the process, with θ denoting the incidence angle of the signal MW field relative to the z-axis, i.e. for optical-bias $\Theta_\theta = |E_p|^2 E_{C1} E_{C2}^* E_D^* \mathcal{E}(\theta)$, where $\mathcal{E}(\theta)$ is a plane wave propagating at angle θ to z-axis. For large beams this simply becomes $\Theta_\theta \approx \exp\left(-5\rho^2/w_0^2 - i\delta k_z(\theta) z\right)$. In Sup. Fig. 6 we plot the power reception pattern $|\eta(\theta)|^2$ normalised to the collinear case $\theta = 0°$ obtained by evaluating the formula Eq. (1) for our experimental conditions.

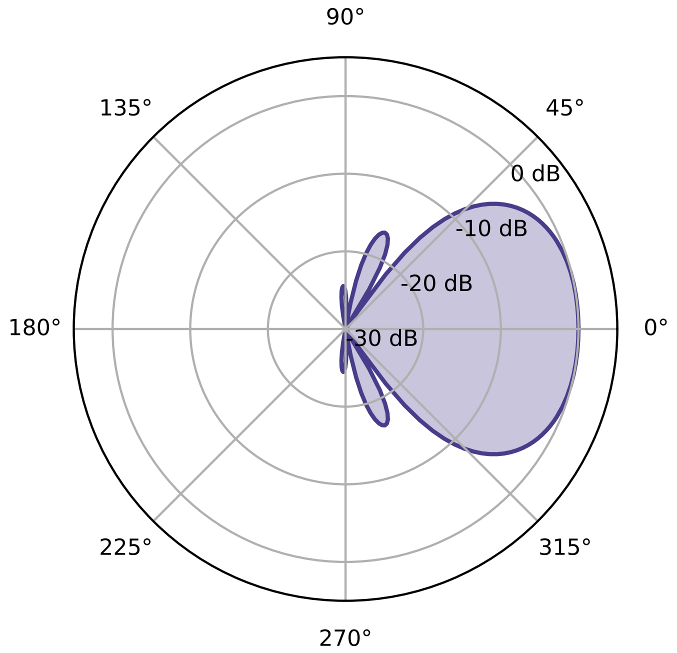

Supplementary Figure 6. Angular dependence of the relative power reception efficiency normalised to collinear configuration corresponding to 0°. The pattern corresponds to an antenna gain of 10 dBi.

[1] Brown, R. C. *et al.* Very-high- and ultrahigh-frequency electric-field detection using high angular momentum rydberg states. *Physical Review A* **107**, 052605 (2023). URL http://dx.doi.org/10.1103/PhysRevA.107.052605.
[2] Wallin, A. E. Allantools (2023). URL https://allantools.readthedocs.io/en/latest/. Accessed: 2025-06-07.
[3] Borówka, S., Pylypenko, U., Mazelanik, M. & Parniak, M. Continuous wideband microwave-to-optical converter based on room-temperature rydberg atoms. *Nature Photonics* **18**, 32–38 (2023). URL https://doi.org/10.1038/s41566-023-01295-w.
